# Characterization of Antimicrobial Properties of Copper-Doped Graphitic Nanoplatelets

**DOI:** 10.3390/ijms252212414

**Published:** 2024-11-19

**Authors:** Jun-Kyu Kang, Seo Jeong Yoon, Honghyun Park, Seung-Jae Lee, Jaehoon Baek, In-Yup Jeon, So-Jung Gwak

**Affiliations:** 1 Department of Chemical Engineering, Wonkwang University, 460 Iksandae-ro, Iksan 54538, Jeonbuk, Republic of Korea; kangjunkyu4843@gmail.com (J.-K.K.);; 2 Advanced Bio and Healthcare Materials Research Division, Korea Institute of Materials Science (KIMS), 797, Changwon-daero, Seongsan-gu, Changwon-si 51508, Gyeongsangnam-do, Republic of Korea; honghyun61@kims.re.kr; 3 Division of Mechanical Engineering, Wonkwang University, 460 Iksandae-ro, Iksan 54538, Jeonbuk, Republic of Korea; 4 School of Energy and Chemical Engineering, Center for Dimension-Controllable Covalent Organic Frameworks, Ulsan National Institute of Science and Technology (UNIST), UNIST-gil 50, Ulsan 44919, Gyeongsangnam-do, Republic of Korea; 5 Nanoscale Sciences and Technology Institute, Wonkwang University, 460 Iksandae-ro, Iksan 54538, Jeonbuk, Republic of Korea; 6 MECHABIO Group, Wonkwang University, 460 Iksandae-ro, Iksan 54538, Jeonbuk, Republic of Korea

**Keywords:** graphitic nanoplatelet, mechanochemical reaction, C–Cu bond, antimicrobial effect

## Abstract

Recent clinical outbreaks of infectious diseases caused by pathogenic microorganisms, such as viruses, bacteria, and fungi, along with the emergence of unwanted microorganisms in industrial settings, have significantly reduced efficiency. Graphene has recently attracted significant attention as a potential antimicrobial agent because of its low toxicity, ease of production and functionalization, and high solubility in water. The presence of oxygen functional groups allows the interaction of the compound with bacteria and other biomolecules, making it an interesting candidate for antimicrobial therapy. Moreover, integrating graphene into copper coatings has been shown to enhance their antimicrobial properties. However, the implementation of copper–graphene composite coatings is currently limited by the difficulty of uniformly distributing graphene within the copper matrix. Copper (Cu)-doped graphitic nanoplatelets (CuGnPs), one option to overcome this challenge, are made via a mechanochemical reaction between solid graphite and Cu powder. The configuration of C–Cu bonds within CuGnPs can be identified using a range of analytical techniques, including transmission electron microscopy, X-ray photoelectron spectroscopy, energy-dispersive X-ray spectroscopy, scanning electron microscopy, and time-of-flight secondary ion mass spectrometry. To evaluate the antibacterial activity of the Cu-GnPs, we employed *Escherichia coli* or Staphylococcus aureus. Various amounts (250, 500, 750, and 1000 μg/mL) of prepared CuGnP samples were incubated in a bacterial suspension for 3 or 6 h at 150 rpm and 37 °C for a colony-forming unit assay. Three hours and six hours of treatment of the bacteria with CuGnPs led to a significant difference in bacterial survival compared with that of the control. It was observed that CuGnPs, with copper bound to graphene oxide, more effectively inhibited the proliferation of *E. coli* compared with nanoplatelets containing graphene oxide alone. These findings suggest that the unique properties of CuGnPs, such as C–Cu bonds, high surface area, and the coexistence of micropores and mesopores, are valuable for exerting strong antimicrobial effects making CuGnPs effective at preventing bacterial colonization on industrial surfaces.

## 1. Introduction

Antibacterial materials are used extensively in daily life to safeguard public health. Various antimicrobial substances, including metal ions and antibiotics, are used to enhance the antimicrobial properties of medical devices such as catheters, implants, and surgical instruments, as well as everyday items such as food packaging [1,2]. These agents work by inhibiting or eliminating pathogens including bacteria, viruses, and fungi from surfaces, helping to minimize the potential for infections. Recent studies in the field of antibacterial materials have increasingly focused on antibiotic-loaded composites, with various antibiotics such as vancomycin, tobramycin, ciprofloxacin, ampicillin, tetracycline, levofloxacin, ibuprofen, and gentamicin having been successfully incorporated into composites for controlled drug delivery [3,4,5,6].

Despite advances in antibiotics, drug-resistant bacteria pose a severe threat to public health globally [7]. According to estimates by the World Health Organization, antibiotic resistance claims approximately 700,000 lives annually, a situation that is projected to worsen. In this context, research aimed at developing novel antibacterial agents by harnessing the physical properties of chemicals and materials has recently been implemented [8]. Various substances, such as antibiotics, metal ions, and quaternary ammonium compounds, have been shown to inhibit the attachment of microbes to material surfaces and their proliferation there [9,10,11]. However, metal ions and quaternary ammonium compounds pose environmental risks and are associated with complex manufacturing processes and high costs. To overcome these challenges, numerous recent studies have focused on the antibacterial properties of nanomaterials, such as silver nanoparticles, titanium oxide nanoparticles, and carbon nanotubes.

Graphene has recently attracted significant attention as a potential antimicrobial agent because of its low toxicity, ease of production and functionalization, and high solubility in water. The presence of oxygen-containing functional groups such as carbonyl, carboxyl, hydroxyl, and alkoxy within graphene permits the compound to interact with bacteria and other biomolecules, increasing its antimicrobial effects. It has been proposed that graphene and graphene oxide (GO) nanomaterials exert antibacterial effects via various mechanisms because of their sharp edges or corners, which can penetrate bacterial cell membranes [12,13].

Numerous studies have demonstrated the antimicrobial potential of noble metal nanoparticles, such as gold and silver. To enhance their antimicrobial effects, these nanoparticles have been combined with other compounds; however, these metals are more costly compared to copper [14]. Ions released from copper or its oxidized forms (copper (I, II, III) oxides) can effectively destroy a wide range of microorganisms. Copper has synergistic effects with antibiotic drugs such as antifungal agent gatifloxacin and the antitubercular drugs capreomycin and disulfiram [15,16,17]. Thus, there has been a recent intensification of research on the antibacterial properties of graphene integrated into copper materials. Copper is one of the most widely used bactericidal reagents, given its low cost and excellent effects killing and inactivating bacteria [18]. Specifically, cupper induces dysfunction in bacterial membranes by generating reactive oxygen species (ROS), thereby inhibiting bacterial adhesion and proliferation, and biofilm formation [19]. Additionally, Cu is characterized by its ability to achieve rapid sterilization and the minimal development of resistance to it [20]. Indeed, owing to its use as an antibacterial material in healthcare settings, Cu has been registered with the US Environmental Protection Agency as the first solid antibacterial material.

We previously reported a one-step edge-functionalization procedure for graphitic nanoplatelets (GnPs) via a mechanochemical reaction [21]. Building on this previous work, in this study, CuGnPs were prepared with graphite and Cu in a solid state, in which C–Cu bonds were formed. The arrangement of C–Cu bonds into CuGnPs was clearly confirmed by a variety of analytical techniques, such as time-of-flight secondary ion mass spectroscopy (TOF-SIMS), X-ray photoelectron spectroscopy (XPS), and transmission electron microscopy (TEM). We also evaluated the antibacterial activity of the CuGnPs by a colony-forming unit (CFU) assay.

## 2. Results and Discussion

Graphene, consisting of a monolayer of carbon atoms organized in a two-dimensional lattice, is a nanomaterial of exceptional thinness. It demonstrates extraordinary mechanical rigidity and outstanding electron transport characteristics. Various forms of graphene sheets have been actively explored, with novel applications in transistors, solar cells, sensors, and biomaterials. Graphene exhibits potent antimicrobial activity against various pathogenic microorganisms, and graphene and its derivatives (such as graphene oxide and reduced graphene oxide) have been reported to exert antimicrobial effects through multiple mechanisms. Also, graphene and its derivatives are highly suitable for use as filler materials in biopolymers for tissue regeneration. Graphene oxide is non-toxic at concentrations below 50 μg/mL for human cells, making it a suitable additive for the preparation of polymer composite scaffolds for clinical applications [22]. Recent studies have focused significantly on the interactions between graphene derivatives and living organisms. Previous studies have reported evaluating the antimicrobial activity of materials composed of simply mixed copper and graphene [23,24,25]. This approach faces challenges in achieving a uniform distribution of copper within the graphene matrix. In this study, we synthesized Copper (Cu)-doped graphitic nanoplatelets (CuGnPs) by binding copper at the edges of graphene and evaluating the antimicrobial efficacy of CuGnPs

**Figure 1:**
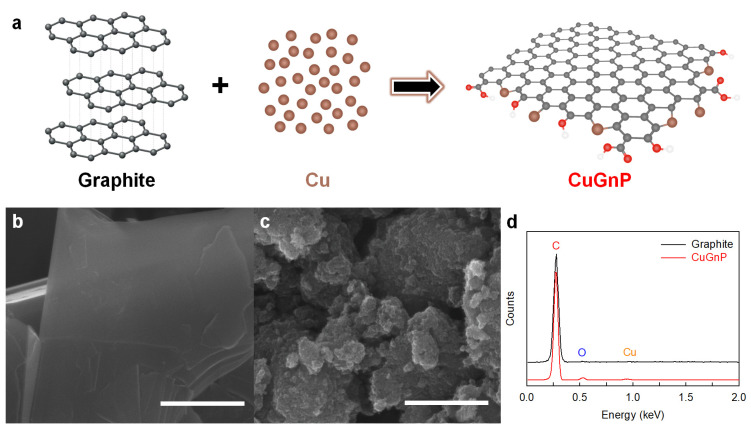
(**a**) Schematic of copper (Cu)-doped graphitic nanoplatelets (CuGnPs) The red circles mean functional groups. Scanning electron microscopy (SEM) images: (**b**) raw graphite and (**c**) CuGnPs. Scale bars represent 1 µm. (**d**) Energy-dispersive X-ray (EDX) spectra.

**Figure 2:**
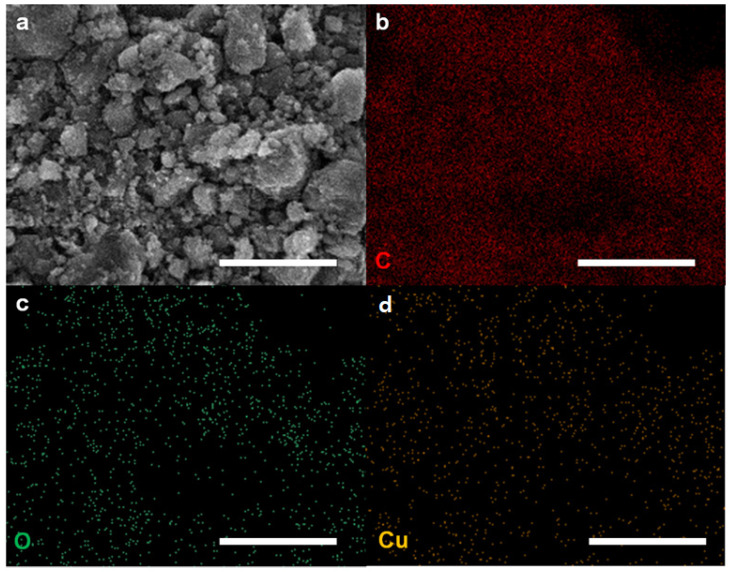
(**a**) Copper-doped graphitic nanoplatelets (CuGnPs). Scale bars represent 1 µm. (**b**) Element mapping images of C for CuGnPs. Scale bar represents 10 µm. (**c**) Element mapping images of O for CuGnPs. Scale bar represents 10 µm. (**d**) Element mapping images of Cu for CuGnPs. Scale bar represents 10 µm.

As shown in Figure 1a, C–Cu bonds were generated between ball milling (i.e., a mechanochemical reaction) with graphite and Cu powder to yield CuGnPs. To remove the unreacted Cu, the resultant CuGnPs were treated with HCl solution. As shown in FE-SEM images, the raw graphite exhibited a flake-like structure with a large particle size (–100 mesh) (Figure 1b). However, after the mechanochemical reaction, the size of the GnPs was dramatically decreased to smaller than several hundred nanometers (nm) (Figure 1c and Figure S1), indicating breakage of the graphitic C–C bonds and the emergence of active carbon species. In addition, owing to the kinetic energy of the metal balls, active Cu species were generated from the broken Cu powder. Thus, the subsequent formation of C–Cu bonds between the active carbon and copper species then led to the arrangement of a snapped graphitic framework and the creation of CuGnPs. In the EDX spectra, CuGnPs displayed a distinct Cu peak with main C and O peaks (Figure 1d), and the Cu content was 0.55 at.% (Table S1). Corresponding element mapping images of C, O and Cu are presented in Figure 2. These results demonstrated that the C–Cu bonds form via a mechanochemical reaction, as shown in Figure 1a.

**Figure 3:**
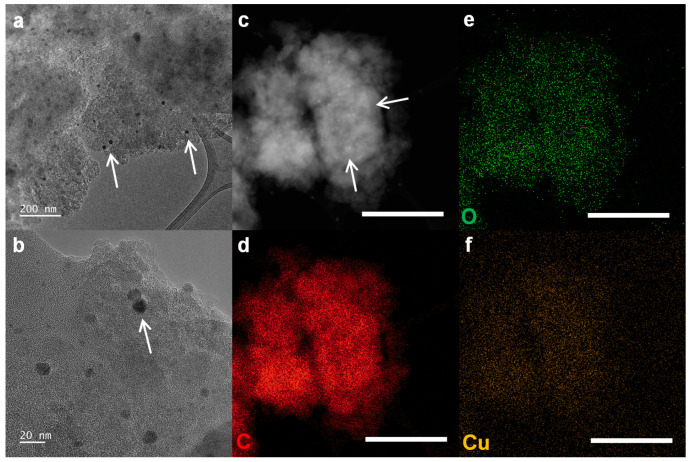
(**a**,**b**) High-resolution transmission electron microscopy (HR-TEM) images of copper-doped graphitic nanoplatelets (CuGnPs), with white arrows indicating Cu nanoparticles. (**c**) High-angle annular dark field (HAADF) scanning transmission electron microscopy (STEM) image of CuGnPs, with white arrows indicating Cu nanoparticles.. (**d**) Element mapping images of C for CuGnPs. Scale bar represents 10 µm. (**e**) Element mapping images of O for CuGnPs. Scale bar represents 10 µm. (**f**) Element mapping images of Cu for CuGnPs. Scale bar represents 10 µm.

Element mapping images for C, O, and Cu are shown in Figure 3d, Figure 33e, and Figure 33f, respectively. HR-TEM was used to visually confirm the presence of Cu into CuGnPs. In the HR-TEM images, the CuGnPs appeared as dark dots corresponding to Cu nanoparticles (white arrows, black dots; Figure 3a,b). A high-angle annular dark field (HAADF) scanning TEM (STEM) image from HR-TEM with Z-contrast showed that the Cu nanoparticles were uniformly distributed without Cu clusters (white arrows, Figure 3a). These images with corresponding element mapping images showed the uniform distribution of Cu nanoparticles in CuGnPs (Figure 3a–c).

**Figure 4:**
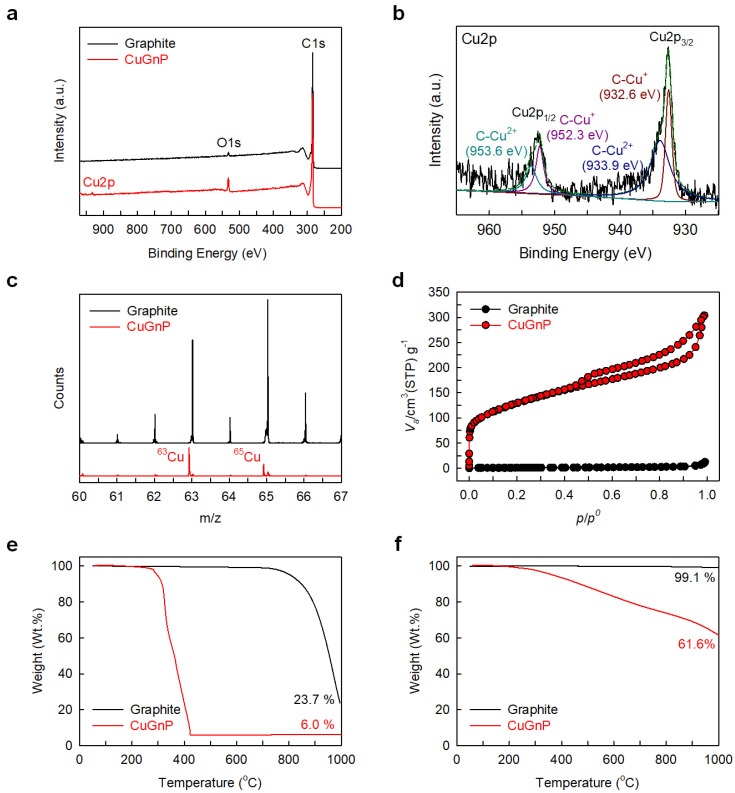
(**a**) X-ray photoelectron spectroscopy (XPS) full-scale survey spectra. (**b**) High-resolution XPS spectra of Cu2p for copper-doped graphitic nanoplatelets (CuGnPs). (**c**) Time-of-flight secondary ion mass spectrometry (TOF-SIMS) spectra. (**d**) N_2_-adsorption/desorption isotherms. (**e**,**f**) Thermogravimetric analysis (TGA) curves of graphite and CuGnPs.

X-ray photoelectron spectroscopy (XPS) can determine the elemental composition of materials. The raw graphite displayed a main C1s peak with a minor O1s peak (Figure 4a), while the CuGnPs displayed a clear characteristic Cu2p peak along with C1s and O1s peaks. Moreover, the Cu content in the CuGnPs was found to be approximately 0.31 at.% (Table S1). In the high-resolution XPS spectra of CuGnPs, the C1s peak was divided into three chemical bonds, namely, C=C (284.4 eV), C-O (285.9 eV), and C=O (289.0 eV) (Figure S2a), while the O1s peak was divided into two chemical bonds, namely, C–O (532.0 eV) and C=O (533.2 eV) (Figure S2b). This could be explained by the unreactive carbon species reacting with the atmosphere (e.g., O_2_ and H_2_O) upon retrieving the samples. Additionally, the Cu2p spectrum was divided into Cu2p_3/2_ and Cu2p_1/2_ peaks (Figure 4b). Cu2p_3/2_ was made up of components at 932.6 and 933.9 eV, while Cu2p_1/2_ had components at 952.3 and 953.6 eV. The former of these peaks at 932.6 and 952.3 eV were related to the C–Cu^+^ bond, while the latter peaks at 933.9 and 953.6 eV were related to the C–Cu^2+^ bond [26].

The successful incorporation of Cu into CuGnPs was clearly identified by TOF-SIMS, in which typical positive-ion mass spectra of the raw graphite and CuGnPs were acquired from a Bi^+^ ion beam. The raw graphite displayed diverse hydrocarbon peaks regardless of Cu (^63^Cu^+^ and ^65^Cu^+^), while the spectrum of CuGnPs clearly showed ^63^Cu^+^ (*m*/*z* = 63) and ^65^In^+^ (*m*/*z* = 65) peaks representing Cu isotopes (Figure 4c). The ^63^In^+^ peak was sharper and more intense than the ^65^Cu^+^ peak because ^63^Cu and ^65^Cu are stable, while ^63^Cu comprises about 69% of the copper present in nature [27].

To measure the specific surface areas (SSAs) of the raw graphite and CuGnPs, the BET method with N_2_ adsorption/desorption isotherms was used (Table S2). Raw graphite with a highly ordered graphitic structure has a very low SSA (2.78 m^2^/g). However, after the mechanochemical reaction, the SSA of CuGnPs (368.57 m^2^/g) was increased 132.6 times compared with that of the raw graphite, indicating delamination into a few graphitic layers due to the edge-selective Cu doping. Based on the theoretical SSA of single-layer graphene (2630 m^2^/g), the mean number of layers of CuGnPs was approximately 7.1 (2630/368 ≈ 7.1). Additionally, CuGnPs displayed both type I (*p*/*p°* = 0–0.5) and type IV (*p*/*p°* = 0.5–1.0) isotherms, indicating the presence of micropores and mesopores respectively (Figure 4d) [28,29]. It is expected that the high surface area along with the coexistence of micropores and mesopores contributes to the enhanced antimicrobial effects of the CuGnPs. These features mean that graphene directly induces mechanical damage to bacterial cell membranes. Specifically, the interaction of bacterial membranes with the surface of graphene confers a tendency for them to tear or for their structure to be disrupted [30]. Moreover, graphene’s high electrical conductivity when combined with copper promotes electron transfer, amplifying its antimicrobial effects [31].

The functionality of CuGnPs was quantitatively analyzed by TGA. The ash yields of the raw graphite and CuGnP at 1000 °C in air were 23.7 and 6.0 wt.%, respectively (Figure 4e. The raw graphite showed high thermal stability due to its large particle size (–100 mesh), but the CuGnPs showed maximum weight loss at approximately 280 °C because of the decomposition of the graphitic structure associated with its very small particle size (<1 µm) and oxygen-containing functional groups. The weight loss of CuGnPs reached a steady state above 420 °C (6.0 wt.%). Considering the ash yields of other GnPs that showed approximately 0.0 wt.% at 1000 °C in air [32,33], the content of CuO or Cu_2_O, which is an amphoteric oxide structure of Cu (Cu oxidizes in air at high temperature), was approximately 6.0 wt.% at 1000 °C. It can thus be calculated that the Cu content in CuGnPs was approximately 4.8–5.2 wt.%. The ash yields of the raw graphite and CuGnPs at 1000 °C in N_2_ were 99.1 and 61.6 wt.%, respectively (Figure 4f).

In the XRD analysis, the raw graphite displayed characteristic peaks at 2θ = 26.5° (002) and 54.7° (004) that are related to the *C*-axis direction perpendicular to the graphite layers [34,35], but CuGnPs showed only a peak at 2θ = 24.1° (002), which drifted by about 2.4° and had only 0.05% of the peak intensity of the raw graphite (Figure S3. The drifted (002) peak with sharply reduced intensity and the vanished (004) peak indicated that most graphitic layers were well exfoliated via the mechanochemical reaction.

**Figure 5:**
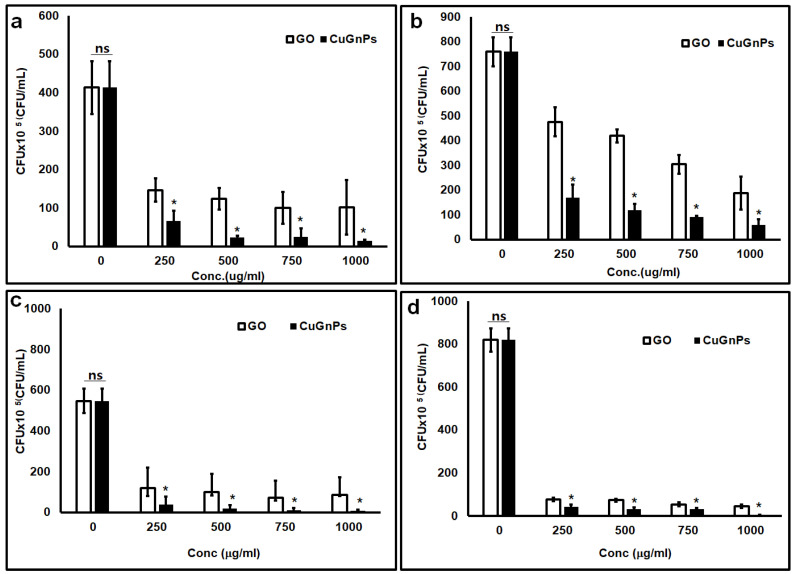
Colony-forming unit (CFU) assay. *Escherichia coli* cultured with CuGnPs for (**a**) 3 and (**b**) 6 h. *Staphylococcus aureus* cultured with CuGnPs for (**c**) 3 and (**d**) 6 h. *: *p* < 0.05 compared to Go nanoplateles. “ns” indicates not significant.

**Figure 6:**
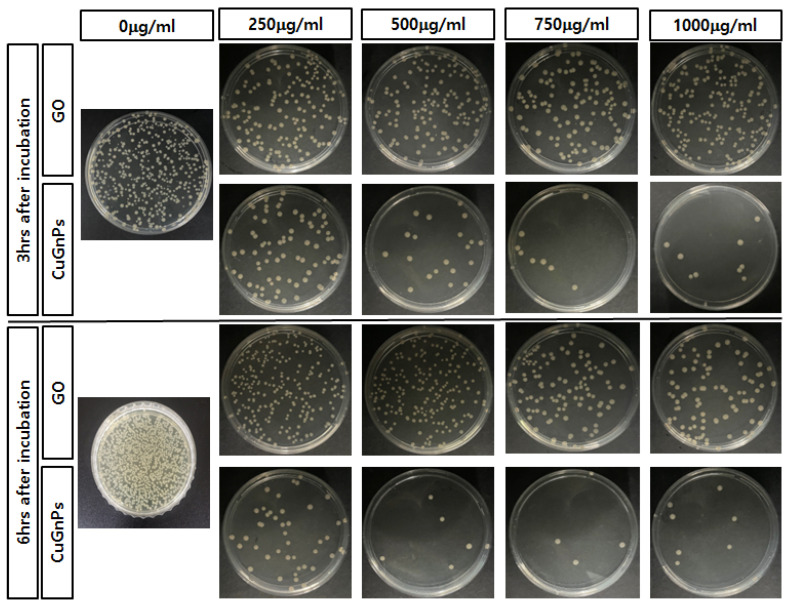
Representative images of viable *E. coli* colonies at 3 h and 6 h post-incubation with GO or CuGNPs at various concentrations.

**Figure 7:**
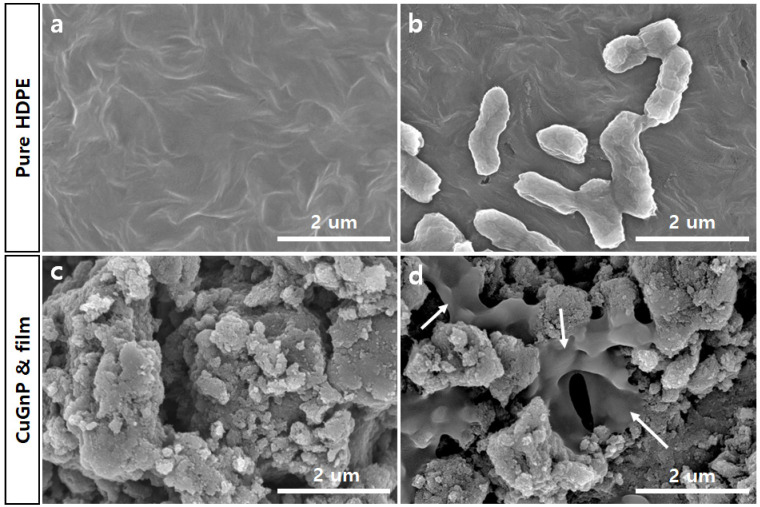
Scanning electron microscopy (SEM) images of (**a**) high-density polyethylene (HDPE) film, (**b**) *Escherichia coli* on HDPE film, (**c**) copper-doped graphitic nanoplatelet (CuGnP) and HDPE film, and (**d**) *E. coli* on CuGnP and HDPE film after 6 h of culture. Arrows indicate membrane damaged *E.coli*. Scale bars represent 2 µm.

This study investigated the antimicrobial effects of CuGnPs against Gram-negative *E. coli* and *S.aureus*. Specifically, the antibacterial efficacy of CuGnPs was determined by a CFU assay. The sterile CuGnP powder was mixed at various concentrations in DI water. These samples were cultured with bacteria at 37 °C for 3 and 6 h. After culture, the bacterial solution was plated in a volume of 20 μL and cultured for 24 h. After 3 h of treatment of the bacteria with CuGnPs, *E. coli* and *S.aureus* exhibited significant differences in survival compared with the control and GO with varying concentrations (Figure 5). As shown in Figure 5, the complexes exhibited much higher activity against both *E. coli* and *S. aureus*. Specifically, the findings demonstrated an approximately 30-fold enhancement of the antimicrobial ability of CuGnPs at 1000 μg/mL for 3 h when compared with the control. The results showed that treatment with CuGnPs induced marked decreases in colony numbers of *E.coli* or *S.aureus* (i.e., from ~85% to ~96% for *E. coli* and from ~76% to ~92% for *S. aureus*), confirming the enhanced bactericidal efficiency of the CuGNPs. Additionally, the group treated with CuGnPs exhibited a significantly greater reduction in CFU compared with the group treated with GO alone, which was probably due to the enhanced antimicrobial effect resulting from the incorporation of Cu into the CuGnPs. For the control group, no significant antibacterial activity is observed up to 6 h, as the number of colony-forming units continuously increases. However, it was observed that colony formation was inhibited over time with a treatment of 1000 ug/mL CuGNPs (Figure 6). Figure 7 shows electron microscopy findings of *E. coli* on film after culture for 6 h. It shows electron microscopy findings of live *E. coli* on the surface of HDPE film. On the HDPE film, it appeared that the membranes were not damaged at all, and intact bacteria were well maintained. Meanwhile, on the surface of the CuGnP and HDPE film, it appeared that the *E. coli* sank because of membrane damage caused by the CuGnPs (Figure 7d). Copper has long been recognized for its antimicrobial properties. In terms of the mechanism involved in this, copper ions (Cu^2+^) penetrate bacterial cell membranes, disrupting their structural integrity and generating ROS intracellularly. These ROS in turn damage intracellular proteins, lipids, and DNA, ultimately impeding bacterial survival [36,37,38]. Similarly, as a result of its nanostructure, graphene causes direct mechanical damage to bacterial membranes. Specifically, when bacteria interact with the surface of graphene, their membranes are vulnerable to tearing and disruption of their structure [39]. Furthermore, graphene’s high electrical conductivity when combined with copper facilitates electron transfer, further enhancing its antimicrobial efficacy [40,41]. In terms of the mechanism behind this additional antimicrobial effect, graphene inactivates bacteria upon contact, which is heavily dependent on the electrical conductivity of the graphene–metal substrate. Consequently, via multiple mechanisms, including sharp-edge cutting, oxidative stress, cell trapping, and encapsulation, bacterial cells that interact with graphene materials are destroyed over time [41,42]. This study confirmed that these mechanisms effectively facilitated the killing of bacteria or the inhibition of their growth.

## 3. Experimental Section

### 3.1. Materials

Graphite was obtained from Alfa Aesar (Natural, −100 mesh, 99.9995% metals basis) and used as received. Copper (powder, <425 µm, 99.5% trace metals basis) was purchased from Aldrich Chemical Inc. (Burlington, MA, USA). and used as received. All other solvents were supplied by Aldrich Chemical Inc. and used without further purification, unless otherwise specified.

### 3.2. Preparation of Copper-Doped Graphitic Nanoplatelets

Copper-doped graphitic nanoplatelets were prepared directly via a mechanochemical reaction (or ball-milling) approach [43]. Graphite (5.0 g) and copper (5.0 g) powder were put into a stainless-steel container containing metal balls of the same materials (500 g, diameter 5 mm). After sealing, atmospheric air was removed using a vacuum pump. The container was then fixed in a planetary ball-mill machine, which was operated at 500 rpm for 48 h. The resultant material was treated with HCl solution to eliminate metallic impurities and unreacted copper. After washing repeatedly with H_2_O, the final product was freeze-dried at −120 °C under reduced pressure (0.05 mmHg) for 48 h to yield 5.53 g (copper content at least 0.53 g) of dark black CuGnP powder.

### 3.3. Analytical Instruments

Field emission scanning electron microscopy (FE-SEM) was performed on FEI Nanonova 230. High-resolution transmission electron microscopy (HR-TEM) was performed on a JEOL JEM-2100F microscope. The TEM specimen was prepared by immersing carbon micro-grids (Ted Pella Inc., Redding, CA, USA 200 Mesh Copper Grid) in well-dispersed samples in ethanol. Time-of-flight secondary ion mass spectrometry (TOF-SIMS) was carried out with a TOF-SIMS V instrument (ION-TOF GmbH, Münster, Germany) using a 10 keV Bi^+^ primary ion beam source. The surface area was measured by nitrogen adsorption–desorption isotherms using the Brunauer–Emmett–Teller (BET) method on Micromeritics ASAP 2504N. X-ray photoelectron spectroscopy (XPS) was performed on a Thermo Fisher K-alpha XPS spectrometer. Themogravimetric analysis (TGA) was conducted on a TA Q200 (TA Instrument) under an air and nitrogen (N_2_) atmosphere at a heating rate of 10 °C/min. X-ray diffraction (XRD) patterns were recorded using a Rigaku D/MAZX 2500V/PC with CuKα radiation (35 kV, 20 mA, λ = 1.5418 Å) at room temperature.

### 3.4. Bacterial Culture

To evaluate the antibacterial activity of the CuGnPs, we employed *Escherichia coli* (*E.coli*, ATCC 25404, ATCC, USC, Manassas, VA, USA) and Staphylococcus aureus (s.aureus, ATCC 25923,ATCC, USC, Manassas, VA, USA). Initially, *E. coli* was cultured in Luria Broth (LB Broth Miller; BD Difco, Franklin Lakes, NJ, USA) in a shaking incubator at 37 °C and 200 rpm until reaching an optical density of 1.7 at 600 nm, as determined using a UV-Vis spectrophotometer (Lambda365; PerkinElmer, Seoul, Republic of Korea).

### 3.5. Evaluation of Antibacterial Activity of Cu-Doped Graphitic Nanoplatelets

The antibacterial activity of the prepared CuGnPs was examined using a CFU assay. The CuGnPs were sterilized by treating them with 70% ethanol for 4 h, followed by centrifugation at 10,000 rpm and 4 °C for 10 min. After centrifugation, the supernatant was removed and the CuGnPs were resuspended in sterilized deionized (DI) water. The bacterial suspension was then diluted with phosphate-buffered saline (PBS, Gibco, Waltham, MA, USA) to an OD_600_ of 1.0 (LB medium:PBS 1:1). Various concentrations of CuGnPs (250, 500, 750, and 1000 μg/mL) were incubated with 2 mL of the diluted bacterial suspension (OD_600_ = 1.0) for either 3 or 6 h at 37 °C and 150 rpm in a shaking incubator. After incubation, 20 μL of the bacterial solution, diluted in LB, was spread on LB agar plates and incubated for 18 h at 37 °C. The bacterial colonies that grew on the agar plates were then counted. Each antibacterial test was performed in triplicate for each condition.

### 3.6. Preparation of Copper-Doped Graphitic Nanoplatelets and High-Density Polyethylene Film

First, high-density polyethylene (HDPE) film was compression-molded at 120 °C and high pressure (30 MPa). The CuGNPs were dispersed in tetrahydrofuran (THF) by tip-sonication for 1 h. The HDPE was covered 10 times with CuGnP/THF solution to prepare the CuGnP and HDPE film, which was then dried at 40 °C for 12 h. As a control, bare HDPE film without CuGnPs was prepared.

### 3.7. Evaluation of Antibacterial Activity of Copper-Doped Graphitic Nanoplatelet Film

After the antibacterial test, the bacteria attached to the CuGnP film surface were observed by SEM. After incubation, the samples were gently washed with PBS and fixed with 4% paraformaldehyde in PBS (PBS, Gibco, Waltham, MA, USA) at room temperature. The fixed samples were washed five times. The samples were then transferred to a new plate and dehydrated through a series of ethanol solutions of 20, 40, 60, 80, and 100 vol% for 15 min. Before observation, the samples with attached bacteria were completely dried and then coated with platinum (5 nm) using a sputtering coater (E-1045; Hitachi, Tokyo, Japan). The CuGnP film was coated by metal sputtering (G20; GSEM, Suwon, Republic of Korea) with 5-nm-thick platinum (Pt) to avoid charging. Then, the coated surface was analyzed by SEM (SU8200 microscope; Hitachi). The SEM images were analyzed by a core facility supporting the analysis and imaging of biomedical materials at Wonkwang University, supported by national research facilities and equipment centers.

### 3.8. Statistical Analysis

To compare three or more groups, a one-way ANOVA or Kruskal−Wallis’s H test was conducted. In all instances, the statistical significance was determined by *p* values of less than 0.05. All quantitative outcomes are presented as the mean ± the standard deviation

## 4. Conclusions

There is a growing demand for public health safeguard products with antimicrobial properties, driven by increasing concerns about infectious diseases and changes in industrial environments. In this study, we fabricated copper-doped graphitic nanoplatelets (CuGnPs) to enhance the antimicrobial properties for applications. Initially, we produced CuGnPs via a mechanochemical reaction with solid graphite and Cu powder. The creation of CuGnPs via the formation of C–Cu bonds was clearly confirmed through diverse analyses, such as TEM, SEM, TOF-SIMS, XPS, and EDX. The coexistence of micropores and mesopores in the CuGnPs, combined with their high and rough surface area, enabled effective interaction with the membranes of *E. coli* and *S.aureus,* damaging them. This in turn reduced the growth of both *E. coli* and *B. subtilis*. In addition, the CuGnPs showed a greater antibacterial effect than the GO nanoplatelets, which was assumed to result from the inclusion of copper in CuGnPs. These findings suggest that the notable features of CuGnPs such as C–Cu bonds, high surface area, and the coexistence of micropores and mesopores enabled substantial antimicrobial effects.

## Data Availability

The data supporting the findings of this study are included within the manuscript.

## Supplementary Materials

The following supporting information can be downloaded at: https://www.mdpi.com/article/10.3390/ijms252212414/s1.
