# Supplementary material for: Characterization of Antimicrobial Properties of Copper-Doped Graphitic Nanoplatelets
Source: Int J Mol Sci. 2024 Nov 19;25(22):12414. doi: 10.3390/ijms252212414 (PMC11594645; doi:10.3390/ijms252212414)
Supplement: Supplementary file 1 [file ijms-25-12414-s001.zip › CUGnP Supplmentary figure.pdf]

Supplemental figure 1

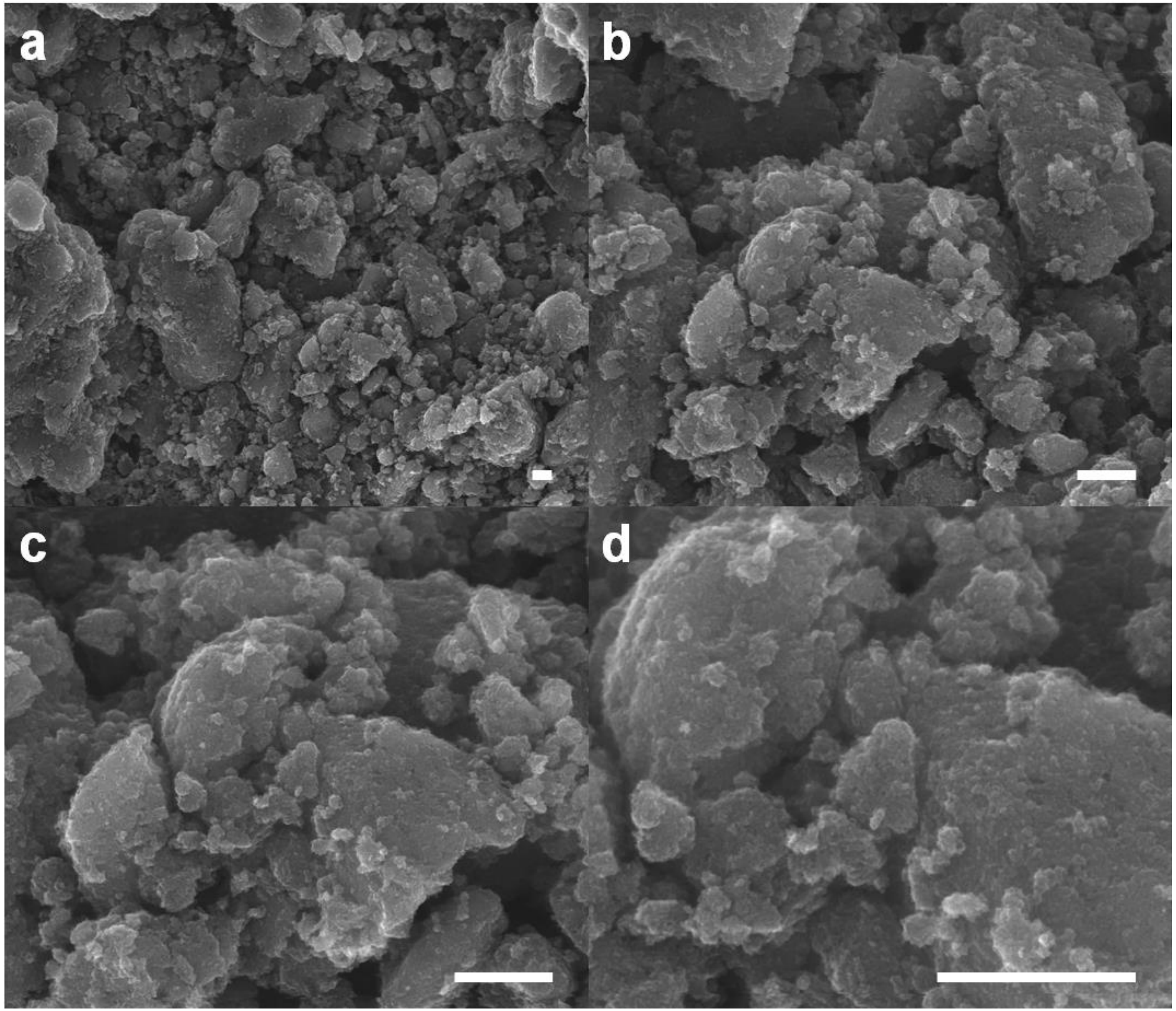

Supplemental figure 2

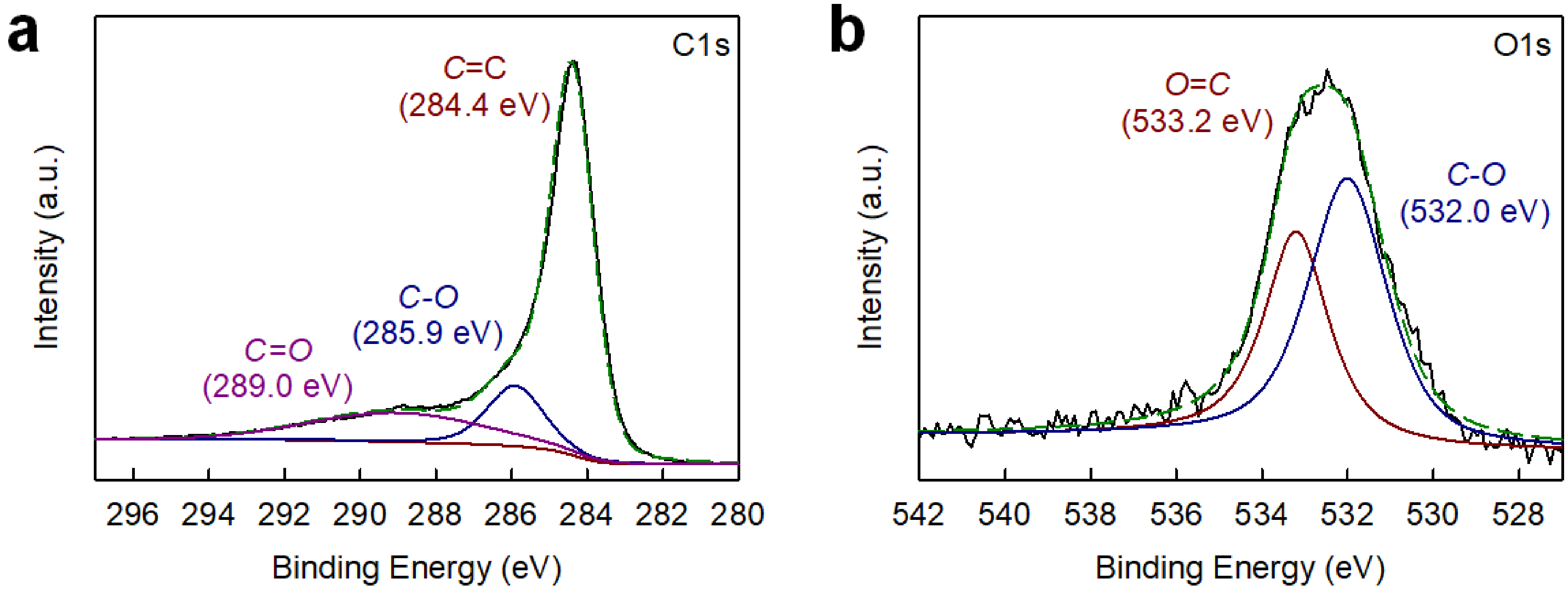

Supplemental figure 3

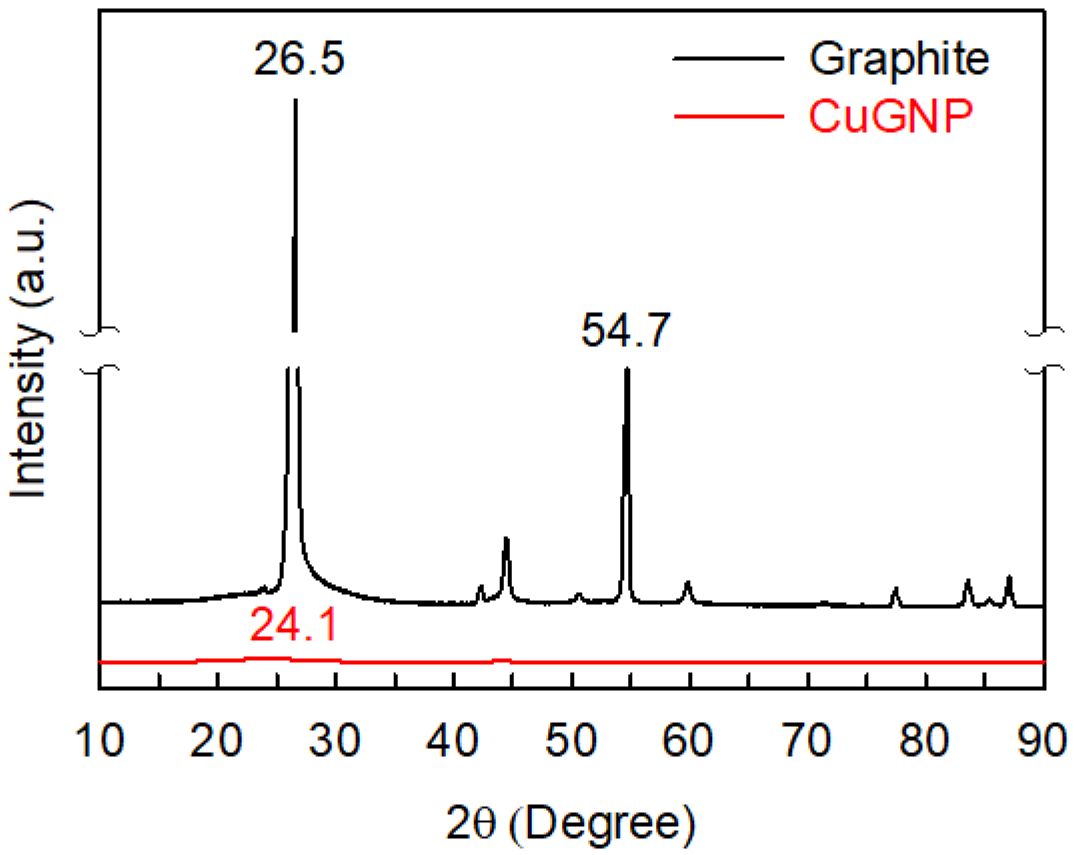

Supplemental figure 4

Sample	TGA (Char yield at 1000 °C)		Element	EDX (wt.%)	XPS (at.%)
	N_2 (%)	Air (%)			
Graphite	99.1	23.7	C (%)	98.80	98.35
			O (%)	1.20	1.65
CuGnP	61.6	6.0	C (%)	95.41	94.50
			O (%)	4.04	5.19
			Cu (%)	0.55	0.31

Supplemental figure 5

Sample	Surface Area (m^2/g)	Pore Volume (mL/g)	Pore Size (nm)
Graphite	2.78	0.0016	2.27
CuGnP	368.57	0.3763	4.08
