# Supplementary material for: Characterization of Antimicrobial Properties of Copper-Doped Graphitic Nanoplatelets
Source: Int J Mol Sci. 2024 Nov 19;25(22):12414. doi: 10.3390/ijms252212414 (PMC11594645; doi:10.3390/ijms252212414)
Supplement: Supplementary file 1 [file ijms-25-12414-s001.zip › Supplemental figure legends.pdf]

Characterization of antimicrobial properties of copper-doped graphitic nanoplatelets

Jun-Kyu Kang[1], Seojeong Yoon[1], Honghyun Park[2], Seung-Jae Lee[3], Jaehoon Baek[4], In-Yup Jeon[1,5,*], So-Jung Gwak[1, 6,*]

[1]Department of Chemical Engineering, Wonkwang University, 460 Iksandae-ro, Iksan, Jeonbuk 54538, Republic of Korea

[2]Advanced Bio and Healthcare Materials Research Division, Korea Institute of Materials Science (KIMS), Republic of Korea

[3]Division of Mechanical Engineering, Wonkwang University, 460 Iksandae-ro, Iksan, Jeonbuk 54538, Republic of Korea

[4]School of Energy and Chemical Engineering/Center for Dimension-Controllable Covalent Organic Frameworks, Ulsan National Institute of Science and Technology (UNIST), UNIST-gil 50, Ulsan, 44919, Republic of Korea

[4]Nanoscale Sciences and Technology Institute, Wonkwang University, 460 Iksandae-ro, Iksan, Jeonbuk 54538, Republic of Korea

[6]MECHABIO Group, Wonkwang University, 460 Iksandae-ro, Iksan, Jeonbuk 54538, Republic of Korea

[*]Corresponding authors: In-Yup Jeon (iyjeon79@wku.ac.kr) and So-Jung Gwak (plus38317@wku.ac.kr)

Supplemental Figures

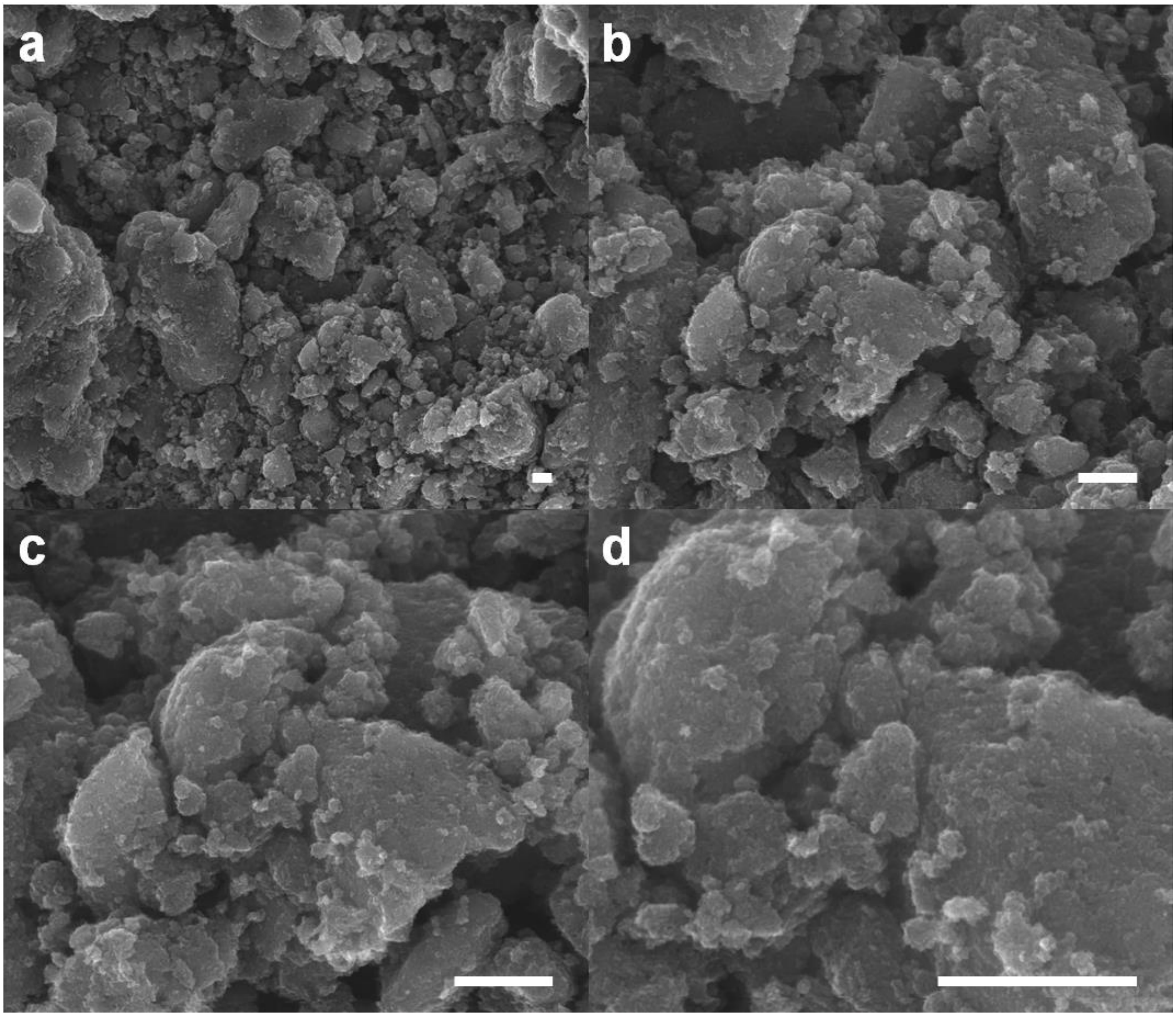

Figure S1. Field emission scanning electron microscopy (FE-SEM) images of copper-doped graphitic nanoplatelets (CuGnPs). Scale bars represent 1 µm.

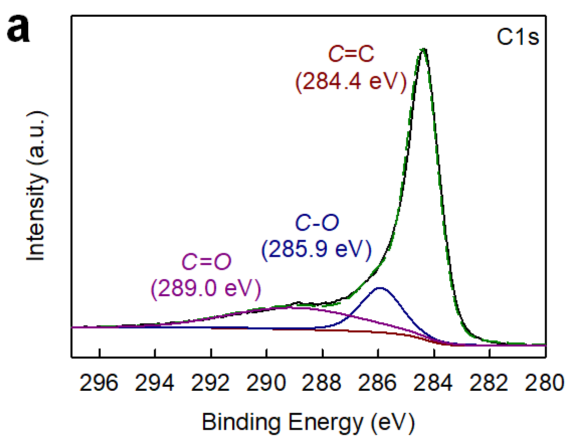

Figure S2. High-resolution X-ray photoelectron spectroscopy (XPS) spectra of copper-doped graphitic nanoplatelets (CuGnPs): (a) C1s and (b) O1s.

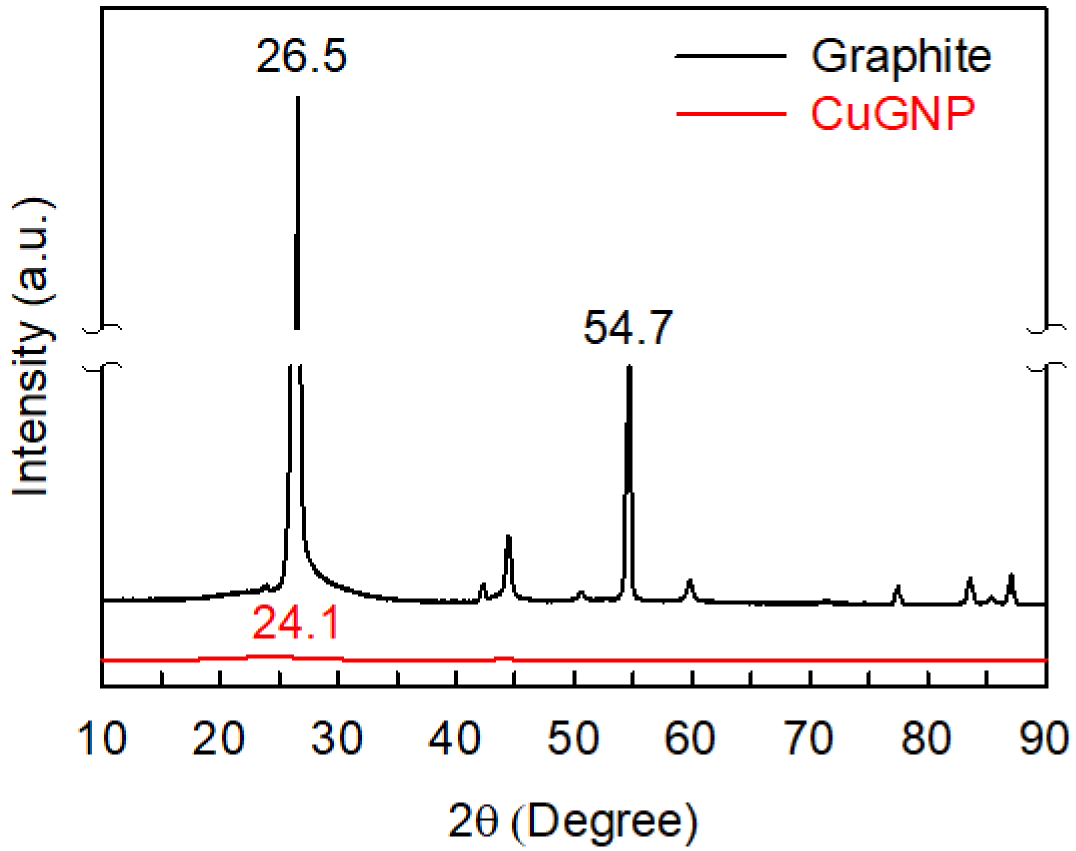

Figure S3. X-ray diffraction (XRD) spectroscopy patterns of the raw graphite and copper-doped graphitic nanoplatelets (CuGnPs).

Sample	TGA (Char yield at 1000 °C)		Element	EDX (wt.%)	XPS (at.%)
	N_2 (%)	Air (%)			
Graphite	99.1	23.7	C (%)	98.80	98.35
			O (%)	1.20	1.65
CuGnP	61.6	6.0	C (%)	95.41	94.50
			O (%)	4.04	5.19
			Cu (%)	0.55	0.31

Table S1. Thermogravimetric analysis (TGA), energy-dispersive X-ray (EDX) spectroscopy, and X-ray photoelectron spectroscopy (XPS) data of the raw graphite and copper-doped graphitic nanoplatelets (CuGnPs)

Sample	Surface Area (m^2/g)	Pore Volume (mL/g)	Pore Size (nm)
**Graphite**	**2.78**	**0.0016**	**2.27**
**CuGnP**	**368.57**	**0.3763**	**4.08**

Table S2. Brunauer–Emmett–Teller (BET) surface area, pore volume, and pore size of the raw graphite and copper-doped graphitic nanoplatelets (CuGnPs)
